# Screen Time Before 2 Years of Age and Risk of Autism at 12 Years of Age

**DOI:** 10.1001/jamapediatrics.2024.4432

**Published:** 2024-11-04

**Authors:** Ping-I. Lin, Weng Tong Wu, Yue-Liang Leon Guo

**Affiliations:** 1 Department of Psychiatry and Behavioral Neuroscience, Saint Louis University School of Medicine, St Louis, Missouri; 2 School of Clinical Medicine, University of New South Wales, Sydney, New South Wales, Australia; 3 Department of Environmental and Occupational Medicine, National Taiwan University (NTU) and NTU Hospital, Taipei, Taiwan

## Abstract

This cohort study evaluates the association between screen time and autism spectrum disorder by considering socioeconomic factors as instrumental variables.

Accumulating evidence suggests that screen time during early childhood (by 5 years of age) may be associated with risk of autism spectrum disorder (ASD), although the causal relationship remains elusive.^1^ One recent study reported a positive association between risk of ASD and length of screen time based on a sample consisting of 84 030 children.^2^ Another study suggests that daily screen time of 8.5 hours or longer was associated with diagnosis of ASD.^3^ However, such an association was not confirmed in other populations,^4^ possibly because of unmeasured confounders. To address these challenges, this study evaluates the association between screen time and ASD by considering socioeconomic factors as instrumental variables.

## Methods

Cohort data from the Longitudinal Study of Australian Children were analyzed. The outcome variable was assessed between 2016 and 2018. High stability of parent-reported ASD diagnosis across multiple waves has been documented.^5^ Early childhood screen time was defined as the weekly number of hours of exposure to television, videos, or other internet-based programs at 2 years of age. Multivariable logistic regression models were used to determine whether early childhood screen time was associated with risk of ASD at 12 years by adjusting for sex, family income, and maternal education level. The instrumental variable method was then used to interrogate the association between early childhood screen time and risk of ASD by assuming that some socioeconomic factors can only directly be associated with screen time instead of ASD risk (eFigure in Supplement 1). All statistical analyses were performed using Stata, version 18, and the study followed the STROBE reporting guideline. The Human Research Ethics Committee at University of New South Wales waived institutional review board approval because the current analysis used deidentified and publicly available data. Statistical significance was set at 2-sided *P* = .05 using the Wald test.

## Results

The sample consists of 5107 children from the birth cohort in Australia, with a weighted sample size of 243 046. A total of 145 children had a parent-reported diagnosis of ASD at 12 years. Bivariate analysis results indicate that screen time was significantly positively associated with diagnosis of ASD (adjusted odds ratio [AOR], 1.83; 95% CI, 1.26-2.59; *P* = .001), male sex (AOR, 1.31; 95% CI, 1.14-1.50; *P* < .001), lower maternal education attainment (AOR, 1.54; 95% CI, 1.39-1.85; *P* < .001), and lower family income (AOR, 1.49; 95% CI, 1.30-1.71; *P* < .001). Race and ethnicity and other perinatal covariates were not found to be associated with screen time; hence, they were not included in the models. Table 1 summarizes AORs from multivariable logistic regression models, showing that ASD risk was significantly greater in children with more than 14 hours of weekly screen time compared with children with less than 14 hours of weekly screen time by 2 years (AOR, 1.79; 95% CI, 1.24-2.58), adjusting for sex, maternal education, and family income. Maternal education and family income were associated with screen time instead of ASD risk; hence, these 2 variables were selected as instrumental variables. Table 2 summarizes results from the instrumental variable model, showing that screen time was not statistically significantly associated with risk of ASD when both family income and maternal education were treated as an instrumental variable that could only be associated with risk of ASD through screen time.

**Table 1.** Results Showing Association Between Screen Time and ASD Risk After Adjusting for Other Covariates^a^

Outcome: ASD diagnosis	AOR (95% CI)	*P* value
Model 1		
Screen time (continuous)	1.25 (1.05-1.50)	.01
Sex (female vs male)	0.27 (0.18-0.42)	<.001
Family income (>A$60 000 vs <A$60 000)	0.92 (0.64-1.32)	.64
Maternal education (>13 y vs <13 y)	1.49 (0.97-2.29)	.07
Model 2		
Screen time (>14 h vs <14 h)	1.79 (1.24-2.58)	.002
Sex (female vs male)	0.27 (0.18-0.42)	<.001
Family income (>A$60 000 vs <A$60 000)^b^	0.92 (0.64-1.32)	.66
Maternal education (>13 y vs <13 y)	1.50 (0.98-2.31)	.06

Abbreviations: ASD, autism spectrum disorder; AOR, adjusted odds ratio.

^a^ Screen time was treated as a continuous variable and a dichotomous variable in model 1 and model 2, respectively.

^b^ Annual income of A$60 000 (US $40 794), a median income, was chosen as the cutoff.

**Table 2.** Results From Instrumental Variable Models Showing Association Between Screen Time and ASD Risk After Controlling for Sociodemographic Factors^a^

Outcome variable: ASD risk	Coefficient (95% CI)	*P* value
**Model 1**		
Screen time (continuous)	−0.30 (−0.79 to 0.18)	.22
Sex (female vs male)	−0.55 (−0.73 to −0.38)	<.001
Variables influencing screen time		
Family income (>A$60 000 vs ≤A$60 000)^b^	−0.14 (−0.22 to −0.07)	<.001
Maternal education (>13 y vs ≤13 y)	−0.24 (−0.22 to −0.17)	<.001
Confounder: sex (female vs male)	−0.10 (−0.17 to −0.03)	.005
**Model 2**		
Screen time (>14 h vs ≤14 h)	−0.73 (−1.90 to 0.45)	.23
Sex (female vs male)	−0.54 (−0.73 to −0.36)	<.001
Variables influencing screen time		
Family income (>A$60 000 vs ≤A$60 000)^b^	−0.06 (−0.09 to −0.03)	<.001
Maternal education (>13 y vs ≤13 y)	−0.10 (−0.13 to −0.06)	<.001
Confounder: sex (female vs male)	−0.04 (−0.07 to −0.01)	.008

Abbreviation: ASD, autism spectrum disorder.

^a^ Screen time was treated as a continuous variable and a dichotomous variable in model 1 and model 2, respectively. Please refer to the eFigure in Supplement 1 for details about the instrumental variable models.

^b^ Annual income of A$60 000 (US $40 794), a median income, was chosen as the cutoff.

## Discussion

The major limitation of the study stems from the possibility of residual confounding effects because the validity of the instrumental variable model depends on the strength of the instruments, which may not fully account for unmeasured socioeconomic or environmental factors. Although our findings suggest that the association between screen time and ASD risk is not causal, there are still important clinical and policy implications. Clinicians could inquire about screen time during early childhood as part of a broader assessment of child development. Screen time can be a useful marker for identifying families needing additional support. Interventions should address underlying socioeconomic factors, providing resources to reduce adverse health impacts of screen time.

## Notes

Supplement 1. **eFigure.** The Graph to Illustrate the Instrumental Variable Method to Determine the Causal Relationship Between Screen Time and Risk of Autism Spectrum Disorder (ASD)

Supplement 2.
Data Sharing Statement
