# Supplementary material for: Screen Time Before 2 Years of Age and Risk of Autism at 12 Years of Age
Source: JAMA Pediatr. 2024 Nov 4;179(1):90–1. doi: 10.1001/jamapediatrics.2024.4432 (PMC11536305; doi:10.1001/jamapediatrics.2024.4432)
Supplement: Supplement 1. — eFigure. The Graph to Illustrate the Instrumental Variable Method to Determine the Causal Relationship Between Screen Time and Risk of Autism Spectrum Disorder (ASD) [file jamapediatr-e244432-s001.pdf]

Supplemental Online Content

Lin PI, Wu WT, Guo YLL. Screen time before 2 years of age and risk of autism at 12 years of age. *JAMA Pediatr*. Published online November 4, 2024. doi:10.1001/jamapediatrics.2024.4432

eFigure. The Graph to Illustrate the Instrumental Variable Method to Determine the Causal Relationship Between Screen Time and Risk of Autism Spectrum Disorder (ASD)

This supplemental material has been provided by the authors to give readers additional information about their work.

© 2024 Lin PI et al. *JAMA Pediatrics*.

eFigure. The Graph to Illustrate the Instrumental Variable Method to Determine the Causal Relationship Between Screen Time and Risk of Autism Spectrum Disorder (ASD).

When using the instrumental variable (IV) method to determine the causal relationship between screen time and the risk of autism spectrum disorder (ASD), it is assumed that the IVs, such as maternal education and family income, meet the following criteria: they must be correlated with screen time (relevance); they should not be correlated with the error term in the regression equation, implying no direct effect on ASD risk except through screen time (exogeneity); and there must be no direct path from these IVs to ASD risk other than through screen time (exclusion restriction).

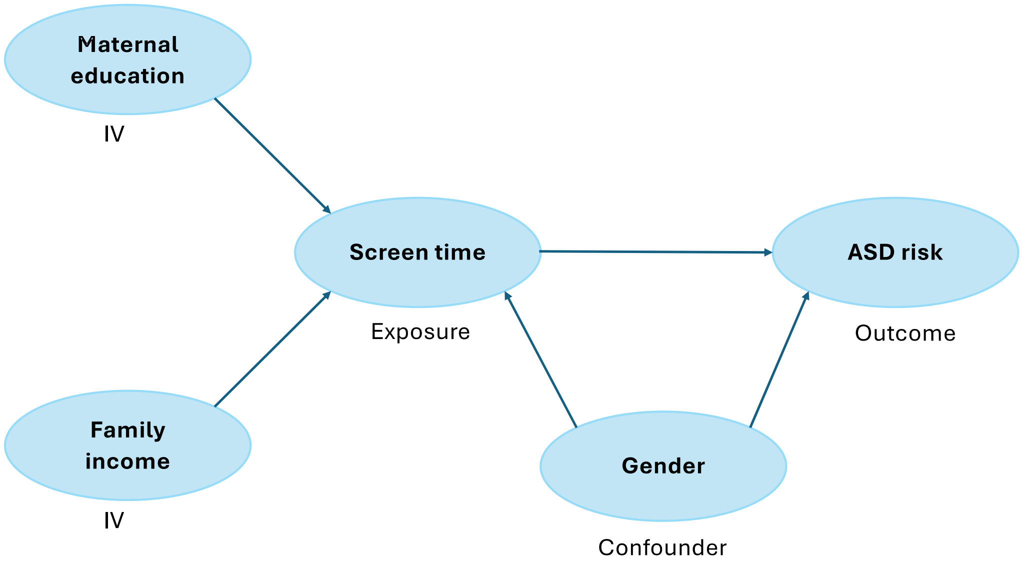

© 2024 Lin PI et al. *JAMA Pediatrics*.
