# Supplementary material for: Screen Time Before 2 Years of Age and Risk of Autism at 12 Years of Age
Source: JAMA Pediatr. 2024 Nov 4;179(1):90–1. doi: 10.1001/jamapediatrics.2024.4432 (PMC11536305; doi:10.1001/jamapediatrics.2024.4432)
Supplement: Supplement 2. — Data Sharing Statement [file jamapediatr-e244432-s002.pdf]

Data Sharing Statement

Lin. Screen Time Before 2 Years of Age and Risk of Autism at 12 Years of Age. *JAMA Pediatr*. Published November 04, 2024. doi:10.1001/jamapediatrics.2024.4432

Data
Data available: Yes
Data types: Deidentified participant data
How to access data: The data access can be requested by submitting an application to "Growing Up in Australia" https://growingupinaustralia.gov.au/.
When available: With publication

Supporting Documents
Document types: None

Additional Information
Who can access the data: Anyone requesting the data
Types of analyses: For any purpose
Mechanisms of data availability: Without investigator support
